# Environmental and Socioeconomic Factors for Gastric Cancer in 14 Counties of the Huai River Basin from 2014 to 2018

**DOI:** 10.3390/ijerph19042213

**Published:** 2022-02-15

**Authors:** Yongqing Lin, Bixiong Ye, Qin Wang, Shaoxia Dong

**Affiliations:** National Institute of Environmental Health, Chinese Center for Disease Control and Prevention, No.7 Panjiayuan Nanli, Chaoyang District, Beijing 100021, China; yongqing0522@163.com (Y.L.); yebixiong@nieh.chinacdc.cn (B.Y.); wangqin@nieh.chinacdc.cn (Q.W.)

**Keywords:** gastric cancer, point density of environmental factors, index of socioeconomic factors

## Abstract

To explore the potential relationship between environmental and socioeconomic factors and the risk of gastric cancer (GC) in the Huai River Basin, the GC incidence rate (GIR) and GC mortality rate (GMR) data from 2014 to 2018 in 14 counties of the Huai River Basin were collected from the Chinese Cancer Registration Annual Report. Environmental and socioeconomic parameters were collected through the Statistical Yearbook. The 14 counties were classified into three groups with low, moderate, and high risk of GC according to the point density of environmental factors (PDF) and index of socioeconomic factors (ISF). Significant differences in GIR and GMR were found among the counties with PDF (χ^2^ = 21.36, *p* < 0.01) and ISF (χ^2^ = 11.37, *p* < 0.05) levels. Meanwhile, significant differences in mortality rate were observed among counties with different PDF (χ^2^ = 11.25, *p* < 0.01) and ISF (χ^2^ = 18.74, *p* < 0.01), and the results showed that the ISF and PDF were increased while the GIR and GMR were decreased. Meanwhile, there was a lag effect between them, and we used two models to explore the lag effects between ISF, PDF and GIR and GMR; the coefficient influence between the ISF lag phase and GIR was −2.9768, and the coefficient influence between PDF and the lag phase on the GIR was −0.9332, and there were both significant impact when there was a probability of more than 95%. The results showed that the higher the ISF and PDF that lags in one stage, the more GIR was reduced, while the impact of the ISF and PDF on lag stage on mortality was not obvious. We used differential GMM to test the results, and also research results were relatively robust. Overall, GIR and GMR decreased with increasing point density of environmental factors and index of socioeconomic factors.

## 1. Introduction

The Huai River Basin (HRB) is located between the Yangtze River Basin and the Yellow River Basin and spans over 1000 km in the Henan, Anhui, Jiangsu, and Shandong provinces. With the aggravation of pollution in the HRB, reports have emerged about villages with high incidences of gastric cancer [1]. Therefore, people have paid increasingly strong attention to gastric cancer in counties of the HRB.

Gastric cancer (GC) has a high prevalence in the world. According to the latest statistics released by the International Agency for Research on Cancer, there were approximately 608,000 new cases of GC and 433,800 GC deaths worldwide in 2020 [2]. The GC incidence rates (GIRs) and GC mortality rates (GMRs) were significantly higher in East Asia (e.g., Mongolia, Japan, and South Korea) compared to in Northern Europe [3,4]. GC was the third most common malignant tumor in China and one of the primary disease burdens faced by Chinese residents [5]. The detrimental effects of GC are mainly physical and psychological [6]. Furthermore, the development of GC quickly occurred. Finally, most families cannot afford the high cost of treatment; therefore, people have paid an intensive attention to the GC.

However, due to economic development, the incidence rates of some cancers have decreased. In recent years, the incidence and mortality of gastric cancer in the Huai River Basin have been constantly changing and have shown a downward trend, which shows that the health management of the Huai River Basin in the past ten years has played a very significant role. In 2014, the top five deaths from malignant tumors in Xuyi County were lung cancer, esophageal cancer, liver cancer, gastric cancer, colorectal cancer, and anal cancer. Gastric cancer ranked fourth, with a crude death rate of 26.74/100,000, which declined compared to 2012 [7,8]. All counties above showed a downward trend in gastric cancer mortality. Researchers of a mortality trend analysis in Sheyang County from 2013 to 2017 found that liver cancer, gastric cancer, and esophageal cancer all showed a downward trend, of which gastric cancer had the largest decline [9].

Having knowledge of the impact of GC on public health, it is essential to improve the understanding of which factors at which point are relevant to the disease. The multifactorial nature of GC mainly includes that it is caused by a combination of genetic and environmental factors, Helicobacter pylori infection, socioeconomic status of one area, diet, and psychology [10,11].

The risk factors for GC vary by region. In the HRB, instances of water pollution were reported numerous times over the past few decades. Studies on the correlation between GC mortality and environmental pollution have revealed a clear relationship between water pollution and digestive tract cancer [12]. The control of wastewater load is still a significant issue for HRB. Pollutant emission is still the major cause of water pollution in the HRB. The construction of sewage treatment facilities (pipe networks, treatment plants, etc.) should be further sped up, and the improvement of industries should be strengthened. Strict control and discharge permit management for the total pollutant amount should be carried out. Meanwhile, Ren et al. used geographic information system (GIS) software to establish a spatial association between digestive tract cancer and sewage discharge in the HRB and then used environmental and health data for over 30 years to reveal a connection between digestive tract cancer and sewage pollution [13]. Yin [14] found a strong link between GC and point density of environmental factors. Some highly polluting enterprises (paper-making, tanning, brewing, chemical industry, etc.) should construct sewage treatment facilities as soon as possible or control the pollutant load by modifying their infrastructure, including closing, combining, or transforming some facilities. In addition, agricultural nonpoint source pollution control should also be strengthened.

Despite conceptual acknowledgment of the importance of social environment for cancer [15,16], socioeconomic factors have rarely been considered in cancer research. There were some studies showed that the impacts of socioeconomic factors and GC; Man [17] found that socioeconomic status was the primary risk factor for GC in the main treatment methods, hospital management, adjuvant therapy, and the period of rehabilitation. Moreover, economic status significantly affects GC prevention [18]. Han [19] found that GIR in China decreased with increases in socioeconomic status in the rural population from 1989 to 2014. Recently published studies have presented a strong association between higher social support and improved cancer treatment results, especially for breast cancer incidence and prognosis [20,21]. As all of these mechanisms interact with each other, a precise assessment of the social environment should not be limited to any specific indicator (e.g., education, profession, income, or employment), but should acknowledge the social environment as a whole [22].

In the Huai River Basin, socioeconomic dissimilarities in health (e.g., cancer incidence) have rarely been analyzed; only one study has been performed in the HRB Anhui Province, which studied the awareness, attitudes, and behaviors of cancer prevention-related risk factors among residents [23], These results are consistent with the most recent reports, which state that the more deprived the area, the higher the burden of disease. The aim of our cross-sectional ecological study was to assess the link between the risks of most frequent cancer sites in HRB and selected environmental and socioeconomic variables: sewage treatment plants, garbage treatment plants and garbage dumps, added value of primary industry, added value of secondary industry, savings balance of urban and rural residents, general public budget income, gross industrial output (current price), and number of hospital beds. These variables were selected because they potentially affect health outcomes throughout the life course. To this day, no studies on this topic have been conducted in HRB. In consideration of the above, we used GIS and SAS software to analyze the differences in GIR and GMR among 14 counties in the HRB based on the point density of environmental factors and index of socioeconomic factors. Due to the heterogeneous distribution of environmental exposure and socioeconomic status, the GIR showed significant spatial differences. The geographical distribution of GC in the 14 counties can be used to improve our understanding of disease and be used to help identify high- and low-risk areas [24]. We chose 14 counties as the unit of analysis because the research question was formulated at the area-level, and the main construct investigated (environmental and socioeconomic determinants) is conceptualized as an area-level attribute. Environmental and socioeconomic determinants are conceptualized as a group attribute that affects all individuals living within the community, and the interest is in drawing inferences regarding differences between areas.

## 2. Materials and Methods

### 2.1. Study Area

**Figure 1:**
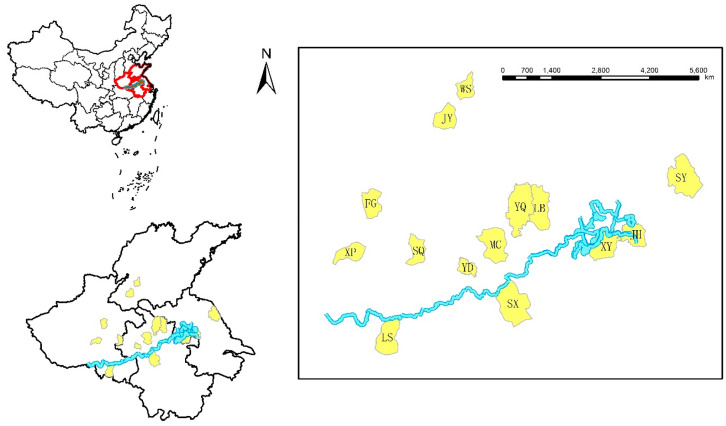
Map of the 14 counties in the HRB.

Fourteen counties belonging to four provinces in the HRB were selected as the study areas: Lingbi (LB), Shouxian (SX), Mengcheng County (MC), Yingdong District (YD), and Yongqiao District (YQ) of Anhui province; Wenshang (WS) and Juye County (JY) of Shandong province; Luoshan (LS), Shenqiu (SQ), Fugou (FG), and Xiping County (XP) of Henan province; and Sheyang (SY), Jinhu (JH), and Xuyi County (XY) of Jiangsu Province. ArcGIS software 10.7 was used to draw the maps shown in Figure 1.

### 2.2. Data Source

The annual GC incidence and mortality data at the county level from 2014 to 2018 were obtained from the Chinese Cancer Registry Annual Report released by the National Cancer Center of China. The indicators included cases and deaths and the GIR and GMR based on the standardization of the Segi’s world population composition, which contains all the populations of the world over 2000 years.

The data used to calculate the Point Density of Environmental Factors came from a survey conducted in the 14 counties of the HRB by the National Institute of Environmental Health, Chinese Center for Disease Control and Prevention from 2012 to 2018. The survey focused on the natural environment, social environment, rural living environment, and sanitation conditions in the 14 counties. In this study, three indicators (harmless disposal points, sewage treatment points, and concentrated stacking points of domestic waste) were selected to represent the efficiency of local sewage and garbage treatment.

Socioeconomic Factors in 2012 to 2018 were obtained from the Chinese Yearbook of Social and Economic Statistics issued by the Chinese Bureau of Statistics. Six county-level indicators were selected in this study to characterize the socioeconomic status in the 14 counties: added value of primary industry, added value of secondary industry, savings balance of urban and rural residents, general public budget income, gross industrial output (current price), and number of hospital beds.

### 2.3. Statistical Analyses

The vectorized county boundary map of the 14 counties in the HRB was taken as the base map in this study. The GIR and GMR values from 2014 to 2018 in the HRB were imported into the GIS system to establish the database. Using ArcGIS 10.7, the Point Density of Environmental Factors was calculated with the GIS spatial connection method. Meanwhile, the Index of Socioeconomic Factors was obtained using SAS 9.4 software based on principal component analysis (PCA). The differences in GIR and GMR values among the counties with Point Density of Environmental Factors and Index of Socioeconomic Factors levels were evaluated using Chi-square tests.

#### 2.3.1. Standardization of GIR and GMR Values

The age structure of the Segi’s world population composition in 2000 was used to standardize the GIR and GMR values in the 14 counties of the HRB from 2014 to 2018. The age-standardized rates were calculated as follows: (1) calculate the age-specific GIRs/GMRs; (2) multiply the age-specific incidence/mortality rates by the corresponding age-structured percentage of the world standard population to obtain the corresponding theoretical incidence/mortality rates; and (3) sum the theoretical age-specific incidence/mortality rates to obtain the age-standardized incidence/mortality rates, as shown in Equation (1):

(1) $$\mathrm{Standardized}\;\mathrm{incidence}\;\mathrm{or}\;\mathrm{mortality}\;\mathrm{rates}=\;\frac{\left(\sum \;\mathrm{standard}\;\mathrm{population}\;\mathrm{age}\;\mathrm{composition}\;\times \;\mathrm{age}-\mathrm{specific}\;\mathrm{incidence}\;\mathrm{or}\;\mathrm{mortality}\;\mathrm{rates}\right)}{\;\left(\sum \;\mathrm{standard}\;\mathrm{population}\;\mathrm{age}\;\mathrm{composition}\right)}$$

where standardized incidence/mortality rates are the GC incidence/mortality rates in one county after standardization in units of 1/100,000; the standard population age composition is the Segi’s world population composition in 2000; age is the entire age structure of one county; and specific incidence/mortality rates are the GC incidence/mortality rates in one county before standardization in units of 1/100,000.

#### 2.3.2. Analysis of Point Density of Environmental Factors

The quantity of the three-point density of environmental factors (sewage treatment plants, garbage treatment plants, and garbage dumps) in a single county was determined using ArcGIS software according to a previously reported method [25,26]. The Point density of environmental factors (*m*), which refers to the number of environmental factors in each county in number/km^2^, was obtained using the Spatial Connection tool in ArcGIS software [27], as shown in Equation (2):

(2) $$m=\frac{\mathrm{Data}\;\mathrm{of}\;\mathrm{environmental}\;\mathrm{factors}\;\mathrm{in}\;\mathrm{the}\;\mathrm{county}}{\mathrm{County}\;\mathrm{Area}},$$

where data of environmental factors in the county is the number of vector planes obtained by longitude and latitude in units of number, and county area is the area of the county in units of km^2^.

#### 2.3.3. Analysis Index of Socioeconomic Factors

The index of socioeconomic factors was evaluated based on six economic factors: the added value of primary industry, the added value of secondary industry, savings balance of urban and rural residents, general public budget income, the total industrial output value (current price), and the number of hospital beds [28]. First, a single factor was normalized as shown in Equation (3):

(3) $$\mathrm{Normalization}\;\mathrm{Index}=\frac{\mathrm{Original}\;\mathrm{value}-\mathrm{Min}\;\mathrm{value}}{\mathrm{Max}\;\mathrm{value}-\mathrm{Min}\;\mathrm{value}},$$

where Normalization index is the normalized value of the factor; Original value is the original value of the factor; Max value is the maximum value of the factor; and Min value is the minimum value of the factor.

After normalization, PCA was carried out at the county level, and the Normalization operation was extracted. Finally, according to the value and contribution of each principal component, Equation (4) was used to calculate the socioeconomic factor scores of each county (SEvalue), with a larger SEvalue indicating a more developed social economy:

(4) $$\mathrm{SEvalue}=F_{1}\frac{{\mathrm{Contribution}}_{1}}{\mathrm{Accumulative}\;\mathrm{Contribution}}+F_{2}\frac{{\mathrm{Contribution}}_{2}}{\mathrm{Accumulative}\;\mathrm{Contribution}}+\ldots F_{3}\frac{{\mathrm{Contribution}}_{3}}{\mathrm{Accumulative}\;\mathrm{Contribution}}+F_{n}\frac{{\mathrm{Contribution}}_{n}}{\mathrm{Accumulative}\;\mathrm{Contribution}},$$

where *F*_1_ through *F_n_* are the principal component values obtained by PCA; Contribution_1_ through Contribution*_n_* are the contribution rates of the central components; and Accumulative Contribution is the total contribution rate of all features.

## 3. Results

### 3.1. GIR and GMR Values in the 14 Counties of the HRB from 2014 to 2018

**Figure 2:**
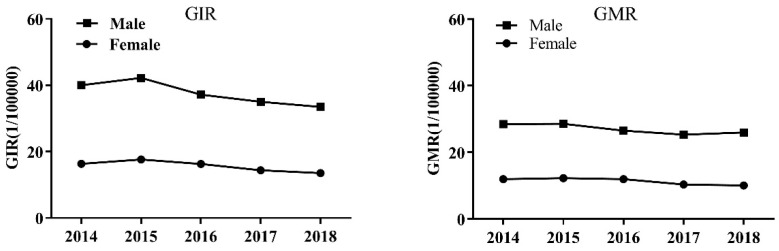
GIR and GMR values from 2014 to 2018.

**Figure 3:**
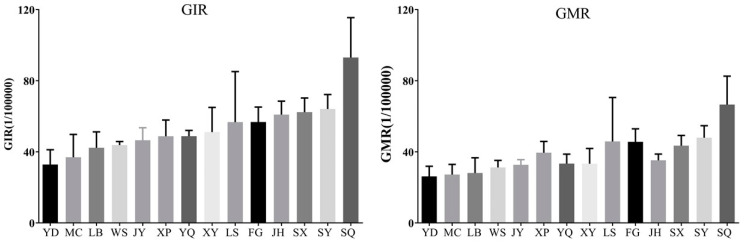
Average GIR and GMR values from 2014 to 2018 in 14 counties.

From 2014 to 2018, the GIR and GMR values in the 14 counties in the HRB showed a decreasing trend over time, and the gender-specific GIR and GMR values showed similar trends in males (Figure 2 and Figure 3).

**Figure 4:**
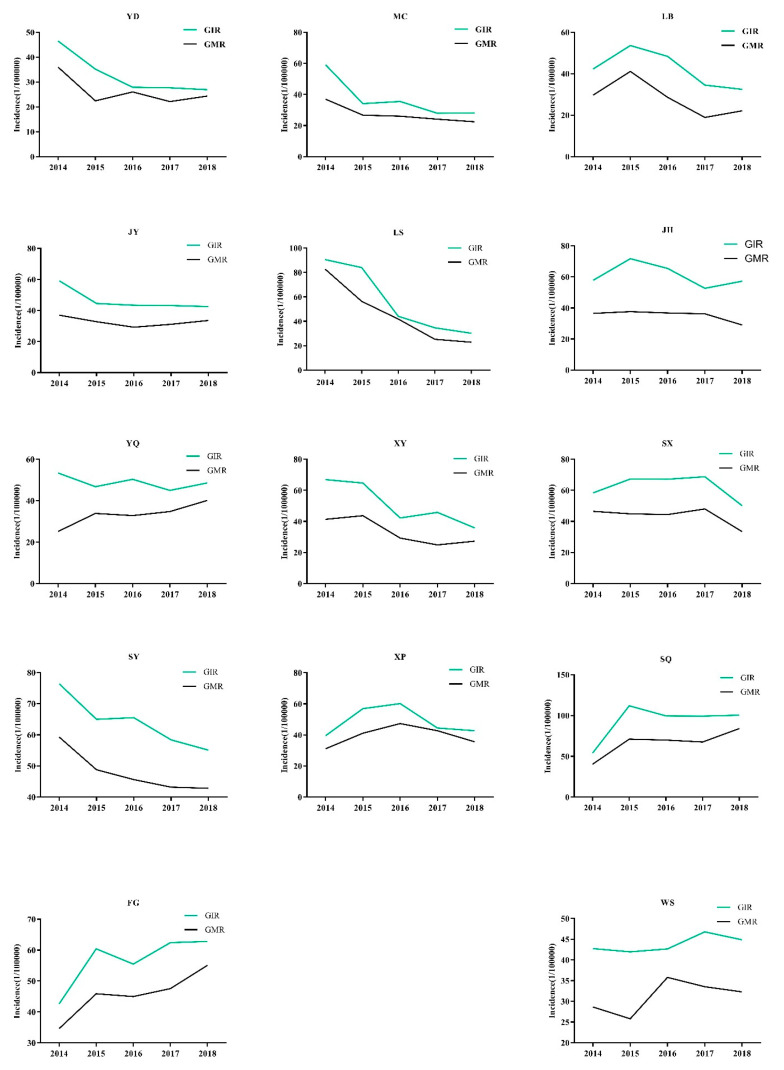
Annual GIR and GMR values from 2014 to 2018 in 14 counties.

As shown in Figure 3, the average GIR and GMR values from 2014 to 2018 in the 14 counties ranged from 32.84/100,000 to 93.05/100,000 and from 26.19/100,000 to 66.55/100,000, respectively. In ten counties (YD, MC, LB, JY, YQ, XY, LS, JH, SX, and SY), GIR decreased over time with annual decreases ranging from 0.20% to 19.73%. In contrast, GIR increased over time in four counties (WS, XP, FG, and SQ). Similarly, the GMRs in the above ten counties decreased over time with annual decreases ranging from 1.91% to 22.7%, while GMR increased in four counties (WS, XP, FG, and SQ), as shown in Figure 4.

### 3.2. Point Density of Environmental Factors

According to the data obtained from the Environmental Factors Survey Project in the 14 counties of the HRB from 2012 to 2018, 1826 pieces of geographical information data related to sewage treatment plants, garbage treatment plants, and garbage dumps were collected for the analysis of point density of environmental factors. Based on the longitude and latitude of the selected environmental point sites, all point information was imported into Arcmap10.7. Combined with the county area of the 14 counties in the HRB, the comprehensive point density of environmental factors was calculated (Table 1).

The point density of environmental factors was then categorized using the following categories: 0 to 0.05 (low), 0.05 to 0.10 (intermediate), and over 0.1 (high). The counties with low values were XY, LS, JY, and JH, SX, SQ the counties with intermediate values were LB, SY, XP, and YQ, and the counties with high values were YD, MC, FG, and WS.

### 3.3. Index of Socioeconomic Factors

Table 2 shows the county-level index of socioeconomic factors values in the 14 counties of the HRB. The index of socioeconomic factors was categorized according to the following categories: 5 to 2 (high), 2 to −1 (intermediate), and −1 to −4 (low). The counties with low values were JY, SQ, LS, and SX, YQ, YD, the counties with intermediate values were FG, XP, MC, and LB, and the counties with high values were XY, SY, WS, and JH.

### 3.4. Interaction between the Point Density of Environmental Factors and Index of Socioeconomic Factors

Based on an analysis using SPSS software, there is no interaction between the point density of environmental factors and the index of socioeconomic factors. Therefore, we only analyzed the relationships between the GIR/GMR and the point density of environmental factors and between GIR/GMR and the index of socioeconomic factors.

### 3.5. Relationships between GIR and GMR with the Point Density of Environmental Factors and Index of Socioeconomic Factors

The differences in GIR and GMR among regions with different Point densities of environmental factors and index of socioeconomic factors values were analyzed by Chi-square tests. Statistically significant differences in GIR were observed between counties with different point density of environmental factors scores (χ^2^ = 21.36, *p* < 0.01) and between counties with different index of socioeconomic factors scores (χ^2^ = 11.37, *p* < 0.05). As shown in Table 3, a higher point density of environmental factors corresponded to a lower GIR. Significant differences were also observed in GMR between counties with different levels of point density of environmental factors and between counties with different levels of index of socioeconomic factors (χ^2^ = 11.25, *p* < 0.01 and χ^2^ = 18.74, *p* < 0.01, respectively). Higher values of point density of environmental factors and index of socioeconomic factors corresponded to lower GMR (Table 4).

## 4. Relevant Tests of Panel Data

In this paper, we carried out the relevant tests of panel data, and after it was processed accordingly, descriptive statistics were carried out to obtain the basic situation of the variable data. The datum was estimated by the Dynamic Panel System, GMM was used to explore the regression result, and then we used the differential GMM estimation method; if the results were still consistent, the model results in this paper were relatively robust.

### 4.1. Build a Model

By setting the explanatory variables as well as the explanatory variables, the model was built as follows

$$GIR_{it}=\beta_{0}+\beta_{1}GIR_{it-1}+\beta_{2}ISF_{it-1}+\varepsilon_{it}\;GMR_{it}=\beta_{\text{0}}+\beta_{1}GMR_{it-1}+\beta_{2}ISF_{it-1}+\varepsilon_{it}\;GIR_{it}=\beta_{0}+\beta_{1}GIR_{it-1}+\beta_{2}PDF_{it-1}+\varepsilon_{it}\;GMR_{it}=\beta_{0}+\beta_{1}GMR_{it-1}+\beta_{2}PDF_{it-1}+\varepsilon_{it}$$

In the above model, *i*: the county (*i* = 1, 2, ..., 14); t: the years (*t* = 2014, 2015, ..., 2018); ε: random error; $\varepsilon_{it}$: other random influencing factors.

### 4.2. Descriptive Statistics

Descriptive statistics were performed on the sample data of each variable to understand the basic situation of the data and to provide a basic understanding of the research data in this article, and the descriptive statistics were shown in Table 5:

The mean GIR was 53.2057, the GMR was 38.3057, the ISF was 0.0003, and the PDF was 0.000017, all of the data are positive numbers, indicating that the average growth rate of the economy was positive or growing.

### 4.3. Dynamic Panel Regression

Since the lag of the interpreted variable may affect the current period of the interpreted variable and solve the endogenous nature that the model might produce, the model was estimated through the Dynamic Panel System, and GMM was used to use the interpreted variable as an instrumental variable to estimate the Dynamic Panel Model, as shown in Table 6:

According to the Arellano–Bond autocorrelation test, if AR (1) rejects the null hypothesis, and the AR (2) test accepts the null hypothesis, it showed that there was no sequence correlation problem in the Dynamic Panel Model, and in the above test, AR (1) corresponds to the *p*-value, the values were 0.0328 and 0.0436, both less than 0.1, and the corresponding *p*-value for AR (2) were 0.9374 and 0.2618, both lager than 0.1, so there was no autocorrelation problem in the model. The probability values corresponding to Sargan values were 0.4746 and 0.7873, and were greater than 0.1, indicating that the acceptance of instrumental variables was not over-recognized, and the setting of instrumental variables was also reasonable. Judging from the above regression results, the interpreted variables of the lag phase had a certain impact on the explanatory variables of the current period, and there was a relatively obvious positive impact; the GIR and GMR of the previous period would affect the GIR and GMR of this period. Otherwise, the coefficient influence between the ISF lag phase and GIR was −2.9768, and the coefficient in-fluence between PDF and the lag phase on the GIR was −0.9332, and there were both significant impact when there was a probability of more than 95%. The results showed that the higher the ISF and PDF that lags one stage, the more GIR was reduced, while the impact of the ISF and PDF on lag stage on mortality was not obvious.

### 4.4. Robustness Testing

The above used the GMM estimation method and then used the differential GMM estimation method; if the results were still consistent, the model results in this paper were relatively robust.

As shown in Table 7, according to the Arellano–Bond autocorrelation test, the AR (1) of the differential GMM corresponds to a *p*-value of 0.0173 and 0.0589, which were less than 0.1, AR (2) corresponds to the *p*-values of 0.9211 and 0.2052, which were greater than 0.1. Therefore, the model did not include autocorrelation issues. The probability values corresponding to the Sargan value were 0.2517and 0.5545, which were larger than 0.1, indicating that the acceptance of the instrumental variable was not over-recognized. It was also reasonable to set the instrumental variables. While the effective coefficient of the ISF and PDF of the one lag phase of the GIR were −2.8586 and −0.9624, there was still a significant negative effect, and the effect of the ISF and PDF of the lag stage on the mortality was not significant, so regardless of the use of GMM or differential GMM, the results obtained were relatively consistent, and the research results were relatively robust.

## 5. Discussion

We investigated the environmental indicators (garbage treatment plants, garbage dumps, and sewage treatment plants) in 14 counties of the HRB from 2014 to 2018. As the point density of environmental factors increased, the GIR and GMR decreased. This suggests that the risk of GC might be related to the point density of environmental factors; as the treatment of sewage and garbage was improved, the GIR and GMR declined. Meanwhile, we collected county-level economic factors (added value of the primary industry, added value of secondary industry, savings balance of urban and rural residents, general public budget income, gross industrial output value (current price), and number of hospital beds) in the 14 counties of the HRB in 2012 to 2018. We found that economic growth was associated with an improvement in people’s living standards. For example, economic development can improve family hygiene, facilitate healthy eating habits, and allow refrigerator use to reduce the risk of GC. In this study, as the index of socioeconomic factors increased, the GIR and GMR values decreased. Torre et al. reported that the improved social security benefits and financial strength in recent years had also provided some guarantees for local cancer treatment [29].

The lack of effective waste treatment might lead to scattered landfills or the burning of garbage, which would contribute significantly to environmental pollution. In addition, the relatively high GIR and GMR values in males might be due to a lack of knowledge about the disease and greater exposure to bacterial infection and other occupational risk factors compared with females [23].

Tian et al. reported digestive tract cancer in middle- and high-risk areas according to the classification of GC and analyzed the relationship between the GC and centralized waste and litter treatment; they also found that the higher GIR, the more waste and litter centralized equipment [30]. Wei et al. showed that untreated wastewater contains many nitrate carcinogens [31]. Meanwhile, wastewater contains many nitrogen-containing compounds. Without effective wastewater disposal, these compounds would be converted into carcinogenic nitrate compounds under the action of various microorganisms. In 2016 years, the Huai River Commission of the Ministry of Water Resources inspected 2139 sewage outlets of rivers and imposed pollution restrictions on several enterprises. The Huai River Water Resources Commission of the Ministry of Water Resources is a full-time organization for comprehensive water resources planning, treatment, and development in the HRB [32]; therefore, the conduction of it had a significant impact on the local disease control.

The socioeconomic conditions also affect malignant tumors. The incidence of GC is higher in areas with relatively low socioeconomic status [12,16]. Vries reported that areas with a monthly income of less than 1000 Won had a higher risk of GC than those with a monthly income higher than 5000 Won [33]. Thus, improvement in the local economy is expected to reduce the occurrence of GC. Developing a better lifestyle, including savings balance of urban and rural residents, general public budget income, and the total industrial output value (current price) in an area could also reduce GC risk and increase the disease rates. The trend in high incidence of gastric cancer among low poverty populations may be explained by a greater participation of the members of wealthier socioeconomic classes in screening programs. This suggests that socioeconomic factors cannot be considered as a determining factor of cancer in a biological sense but since several cancer risk factors are associated with social economics, poverty is a “cause of the cause”.

Finally, we also discussed the lag effect in this study. Judging from the above regression analysis, the model results of GIR and GMR were 0.8920 and 0.9795 respectively, indicating that there was a significant effect by more than 99% probability, which means that the interpreted variables of the lag phase do have a certain impact on the explanatory variables of the current period, and there was a relatively obvious positive relationship that the GIR and GMR of the previous period would affect the GIR and GMR of this period. Meanwhile, the higher the ISF and PDF that lags one stage, the incidence was reduced, while the impact of the economic lag stage on mortality was not obvious. Therefore, we can conclude that the ISF and PDF have one lag effect of gastric cancer’s incidence.

There are some limitations to our study. First, the disease data were extracted from the Chinese Cancer Registration Annual Report, and some data were missing. In the case of missing data, data from neighboring counties were used to make up for the missing values, which may have resulted in information bias. In addition, the economic factors used in this study (e.g., general public budget revenue and the savings balances of urban and rural residents) may not fully represent the income level on the village or family scale. In future studies, other economic variables should be obtained for inclusion in the analysis. The development of digestive tract tumors generally occurs over 10 or 20 years or even longer. The disease data analyzed in this study were from 2014 to 2018, while the point density of environmental factors and index of socioeconomic factors were from 2012 to 2018. Both these time periods are less than 10 years; however, the GIR and GMR values were significantly different between counties and showed the same downward trend over time; meanwhile, the effects of environmental and socioeconomic were accumulated year by year, so the results were still convincing.

We provide the following suggestions for future work. First, more reliable variables should be obtained, including the income status and health intervention measures in villages and households. Second, some spatiotemporal models (e.g., geographically weighted regression and the GeoDetector model) should be used to analyze the spatial heterogeneity of factors influencing GIR and GMR [34].

## 6. Conclusions

In this study, we demonstrated the relationships between PDF/ISF and GIR/GMR. Larger numbers of garbage dumps, garbage treatment plants, and sewage treatment plants were associated with lower GIR and GMR. In addition, as the six evaluated socioeconomic factors (added value of primary industry, added value of secondary industry, savings balance of urban and rural residents, general public budget income, total industrial output value (current price), and number of hospital beds) increased, the GIR and GMR values in the 14 counties of the HRB decreased. Thus, as concrete measures to increase economic investment and improving resident income have taken effect, cancer prevention and control have improved in the HRB, and the better control of environmental sewage and garbage dumps was significant to the GC reduction.

Understanding the associations between different risk factors and GIR/GMR is helpful for identifying interventions to control GC. The findings of this study are valuable for epidemiologists as they provide information on the potential link between cancer development and environmental and economic factors in the HRB. The results suggest that local governments had enhanced measures to improve sewage and garbage treatment to better control GC, and meanwhile the economic were boost increased have decreased the GC.

## Figures and Tables

**Table 1:** Point density of environmental factors in 14 counties of the HRB.

	2012	2013	2014	2015	2016	2017	2018
YD	0.145 ***	0.197 ***	0.142 ***	0.223 ***	0.213 ***	0.252 ***	0.262 ***
MC	0.116 ***	0.238 ***	0.246 ***	0.278 ***	0.241 ***	0.261 ***	0.282 ***
FG	0.138 ***	0.247 ***	0.295 ***	0.291 ***	0.217 ***	0.278 ***	0.377 ***
WS	0.125 ***	0.267 ***	0.269 ***	0.232 ***	0.216 ***	0.231 ***	0.216 ***
LB	0.083 **	0.082 **	0.098 **	0.076 **	0.084 **	0.098 **	0.082 **
SY	0.102 **	0.091 **	0.104 **	0.043 **	0.031 **	0.089 **	0.081 **
XP	0.081 **	0.076 **	0.071 **	0.053 **	0.061 **	0.083 **	0.073 **
YQ	0.101 **	0.071 **	0.072 **	0.091 **	0.081 **	0.092 **	0.074 **
XY	0.055 *	0.042 *	0.051 *	0.012 *	0.035 *	0.031 *	0.035 *
LS	0.027 *	0.026 *	0.029 *	0.013 *	0.014 *	0.017 *	0.034 *
JY	0.029 *	0.021 *	0.025 *	0.042 *	0.015 *	0.043 *	0.015 *
JH	0.017 *	0.043 *	0.027 *	0.025 *	0.031 *	0.042 *	0.031 *
SX	0.064 *	0.031 *	0.035 *	0.039 *	0.015 *	0.018 *	0.015 *
SQ	0.022 *	0.022 *	0.011 *	0.013 *	0.047 *	0.041 *	0.047 *

***: High point density of environmental factors, >0.1; **: median point density of environmental factors, 0.05~0.1; *: low point density of environmental factors, 0~0.05.

**Table 2:** Index of socioeconomic factors for the 14 counties in the HRB.

County	2012	2013	2014	2015	2016	2017	2018
XY	2.15 ***	2.04 ***	2.8 ***	2.1 ***	2.94 ***	3.09 ***	3.13 ***
SY	2.22 ***	4.29 ***	3.46 ***	2 ***	2.33 ***	2.74 ***	3.14 ***
WS	3.1 ***	3.82 ***	3.01 ***	4.1 ***	4.41 ***	4.16 ***	3.04 ***
JH	3.31 ***	3.79 ***	3.23 ***	4.06 ***	3.98 ***	3.96 ***	3.16 ***
FG	0.07 **	−0.19 **	−0.16 **	0.39 **	0.3 **	−0.36 **	−0.29 **
XP	0.16 **	−0.72 **	0.75 **	−0.17 **	−0.37 **	−0.73 **	−0.79 **
MC	0.09 **	−0.48 **	0.02 **	−0.4 **	−0.42 **	0.34 **	0.32 **
LB	0.05 **	−0.98 **	−0.64 **	−0.72 **	−0.65 **	−0.83 **	−0.81 **
JY	−1.62 *	−2.43 *	−1.56 *	−1.76 *	−1.66 *	−1.65 *	−1.75 *
SQ	−2.22 *	−1.18 *	−1.27 *	−1.2 *	−1.22 *	−1.33 *	−1.35 *
LS	−1.17 *	−1.16 *	−1.4 *	-2.09 *	−2.24 *	−1.43 *	−1.47 *
SX	2.49 *	−2.63 *	−1.42 *	−2.1 *	−2.39 *	−1.53 *	−1.71 *
YQ	−2.48 *	−1 *	−2.43 *	−1.8 *	−1.25 *	−2.59 *	−2.6 *
YD	−3.81 *	−1.82 *	−1.58 *	−1.02 *	−2.07 *	−2.87 *	−2.82 *

***: High index of socioeconomic factors, 5 to 2; **: intermediate index of socioeconomic factors, 2 to −1; *: low index of socioeconomic factors, −1 to −4.

**Table 3:** GIR in different groups of counties in the HRB classified by the point density of environmental factors and index of socioeconomic factors from 2014 to 2018.

	2014	2015	2016	2017	2018	5Y ^	χ^2^	*p*
Point Density of Environmental Factors								
Low value area Median value area	65.77 53.87	74.5 58.8	59.82 57.96	58.37 46.98	51.8 47.2	62.63 52.38	21.36 **	<0.01
High value area	47.72	42.92	40.4	41.2	40.7	43.27		
Index of Socio-economic Factors								
Low value area	60.15	64.58	58.33	50.32	47.70	55.58	11.37 *	<0.05
Median value area	57.63	64.45	61.40	48.36	42.89	54.95		
High value area	54.53	60.03	47.66	44.61	42.58	49.32		

^: indicates the average incidence rate over five years. **: *p* < 0.01, *: *p*<0.05.

**Table 4:** GMR in different groups of counties in the HRB classified by the point density of environmental factors and index of socioeconomic factors from 2014 to 2018.

	2014	2015	2016	2017	2018	5Y ^	χ^2^	*p*
Point Density of Environmental Factors								
Low value area Median region	49.57 36.35	49.75 40.5	42.95 38.22	39.38 35.16	40.23 33.93	44.37 36.83	11.25 **	<0.01
High value area	34.04	30.2	33.22	31.84	33.53	32.56		
Index of Socio-economic Factors								
Low value area	39.82	46.67	49.65	46.75	48.67	46.31	18.74 **	<0.01
Median region	45.36	40.17	35.17	31.85	34.1	36.79		
High value area	34.3	34.03	31.42	28.19	30.99	31.78		

^: indicates the average mortality rate over five years. **: *p* < 0.01.

**Table 5:** Descriptive statistics.

Variable	Obs	Mean	Std. Dev.	Min	Max
GIR	70	53.2057	18.3246	26.9400	111.9300
GMR	70	38.3057	13.7211	18.9400	84.0300
PDF	70	0.000017	0.05267	0.012000	0.37700
ISF	70	0.0003	1.2359	−1.8000	3.4600

**Table 6:** The Dynamic Panel System GMM estimates.

	Model 1	Model 2
VARIABLES	GIR	GMR
L.GIR	0.8920 **	
	(12.5166)	
L.GMR		0.9795 **
		(8.4945)
L.PDF	−0.9322 * (−2.6349)	1.3256 (2.7612)
L.ISF	−2.9768 *	1.1984
	(−2.3485)	(1.3197)
Constant	3.2980	0.2038
	(0.6965)	(0.0469)
Observations	56	56
Number of id	14	14
AR (1)	0.0328	0.0436
AR (2)	0.9374	0.2618
Sargan (*p*-value)	0.4746	0.7873

**: There was a significant effect more than 99% probability, *: There was a significant effect of more than 95% probability. The absence of an asterisk indicated that there was no significant effect. Inside the parentheses was the *t*-value. L stands for one lag item.

**Table 7:** Differential GMM regression results.

	Model 1	Model 2
VARIABLES	GIR	GMR
L.GIR	0.8725 **	
	(3.8458)	
L.GMR		0.8081 **
		(4.3345)
L.PDF	−0.9624 * (−1.9858)	1.8653 (1.4824)
L.ISF	−2.8586 *	1.1548
	(−1.9859)	(1.0958)
Constant	3.3732	5.3909
	(0.2836)	(0.7322)
Observations	42	42
Number of id	14	14
AR (1)	0.0173	0.0589
AR (2)	0.9211	0.2052
Sargan (*p*-value)	0.2517	0.5545

**: There was a significant effect more than 99% probability, *: There was a significant effect of more than 95% probability. The absence of an asterisk indicated that there was no significant effect. Inside the parentheses was the *t*-value. L stands for one lag item.

## Data Availability

The datasets used and analyzed during the current study are available from the corresponding author on reasonable request.

## Abbreviations

HRB	Huai River Basin
GIS	Geographic Information System
GC	Gastric Cancer
GIR	Gastric Cancer Incidence Rate
GMR	Gastric Cancer Mortality Rate
LB	Lingbi
SX	Shouxian
MC	Mengcheng
YD	Yingdong District
YQ	Yongqiao District
WS	Wenshang
JY	Juye
LS	Luoshan
SQ	Shenqiu
FG	Fugou
XP	Xiping
SY	Sheyang
JH	Jinhu
XY	Xuyi
PDF	Point Density of Environmental Factors
ISF	Index of Socioeconomic Factors
